# High risk behavior for HIV transmission among former injecting drug users: a survey from Indonesia

**DOI:** 10.1186/1471-2458-10-472

**Published:** 2010-08-10

**Authors:** Shelly Iskandar, Diba Basar, Teddy Hidayat, Ike MP Siregar, Lucas Pinxten, Reinout van Crevel, Andre JAM Van der Ven, Cor AJ De Jong

**Affiliations:** 1 Department of Psychiatry, Faculty of Medicine, Padjajaran University/Hasan Sadikin Hospital, Bandung, Indonesia; 2 Health Research Unit, Faculty of Medicine, Padjadjaran University/Dr. Hasan Sadikin Hospital, Bandung, Indonesia; 3 Department of Health Education and Health Promotion, Faculty of Health, Medicine and Life Sciences, Maastricht University, Maastricht, the Netherlands; 4 Department of General Internal Medicine and Nijmegen Institute for Inflammation, Infection and Immunity, Radboud University Nijmegen Medical Centre, Nijmegen, the Netherlands; 5 Nijmegen Institute for Scientist-Practitioners in Addiction (NISPA), Nijmegen, the Netherlands

## Abstract

**Background:**

Injecting drug use is an increasingly important cause of HIV transmission in most countries worldwide, especially in eastern Europe, South America, and east and southeast Asia. Among people actively injecting drugs, provision of clean needles and opioid substitution reduce HIV-transmission. However, former injecting drug users (fIDUs) are often overlooked as a high risk group for HIV transmission. We compared HIV risk behavior among current and former injecting drug users (IDUs) in Indonesia, which has a rapidly growing HIV-epidemic largely driven by injecting drug use.

**Methods:**

Current and former IDUs were recruited by respondent driven sampling in an urban setting in Java, and interviewed regarding drug use and HIV risk behavior using the European Addiction Severity Index and the Blood Borne Virus Transmission Questionnaire. Drug use and HIV transmission risk behavior were compared between current IDUs and former IDUs, using the Mann-Whitney and Pearson Chi-square test.

**Results:**

Ninety-two out of 210 participants (44%) were self reported former IDUs. Risk behavior related to sex, tattooing or piercing was common among current as well as former IDUs, 13% of former IDUs were still exposed to contaminated injecting equipment. HIV-infection was high among former (66%) and current (60%) IDUs.

**Conclusion:**

Former IDUs may contribute significantly to the HIV-epidemic in Indonesia, and HIV-prevention should therefore also target this group, addressing sexual and other risk behavior.

## Background

Worldwide, injecting drug use is estimated to account for just less than one-third of new infections outside sub-Saharan Africa [1]. HIV-prevention programs for injecting drug users (IDUs) therefore put emphasis on people actively injecting drugs, especially through needle exchange or opioid replacement. Besides active IDUs, people who have a previous history of injecting drug use (former IDUs) are probably also an important risk group for HIV transmission. However, relatively little is known about this group. Sporadic studies from western countries have shown that former IDUs (fIDUs) may have a high risk of becoming HIV-infected or spreading HIV to others [2,3]. To our knowledge, no studies on fIDUs have been reported from low- or middle-income countries.

Injecting drug use increased dramatically in the late '90s in Indonesia, acting as the main force driving the HIV-epidemic. Among the general population, the prevalence of HIV-infection is still low (0.3%), but up to 50% or more of IDUs are already HIV-infected [4]. Drug use is illegal in Indonesia, and harm reduction programs, although officially supported by the Indonesian government, only reach a minority of IDUs. Apart from sharing needles, sexual risk behavior is also common among drug users [5]. In three large cities in Indonesia, over two thirds of IDUs were sexually active, of whom many reported having multiple partners (48%) and sex with female sex workers (40%) in the preceding year. Consistent condom use was only reported by 10% of sexually active IDUs [5].

We know of no reported data concerning HIV risk behavior among fIDUs in Indonesia, and fIDUs receive very little attention in current prevention programs in general. This may seriously limit the success of HIV-prevention focusing on drug injection, as a considerable number of IDUs change from injection to non-injection drug administration or completely abstain from illicit drug use [6]. The prevalence of HIV may be high in fIDUs. Transmission of blood borne viruses may continue to occur through sexual behavior and/or by contaminating equipment that is subsequently used by others for drug use, tattooing and/or piercing [2,7,8].

Furthermore, fIDUs may also play an important role in transmitting HIV infections to the general population because, compared with current IDUs (cIDUs), fIDUs have more sexual contact with people who do not use drugs [9,10]. Hopefully, a better characterization of former IDUs may contribute to improve HIV-prevention. Therefore, the aim of the present study was to explore the characteristics and the risk behavior of former IDUs in Indonesia in comparison with current IDUs.

## Methods

### Setting and patients

From June to September 2008, 210 IDUs were recruited in Bandung, the capital of West-Java and epicenter of the epidemic of injecting drug use in Indonesia. Respondent driven sampling, a form of peer recruitment, was used for recruitment of IDUs from the community [11]. With help from local non-governmental organizations involved in outreach to IDUs, three cIDUs and three fIDUs from different parts of Bandung were selected to act as 'seeds' for RDS and invited to a community clinic which has a specific program for IDUs. Following their inclusion in the study, these six seeds were asked to recruit two other persons injecting drugs, either in the last six months (cIDUs) or longer ago (fIDU), by giving individually numbered coupons. IDUs presenting at the community clinic with the coupons, were asked themselves to recruit two other (current or former) IDUs. This process of recruitment continued until the desired sample size was achieved. Numerical simulations have shown that respondent driven sampling estimates converge to the true values even if the seeds are not drawn as desired [12].

As a part of the RDS process, an incentive was offered for participating in the interview ($3) and for recruiting two injecting drug using peers ($2 per eligible peer recruited). After the initial seeds were recruited, only those people who presented coupons were permitted to participate in the study. The study was completely anonymous, but to prevent the same participant from entering the study twice, physical marks such as tattoos, scars, or birth marks were recorded.

Only those candidates who were or had previously been IDUs were eligible to be included in the study. Two outreach workers from non-governmental harm reduction organizations, both with a previous history of drug use, confirmed that the respondents were indeed IDUs. To this purpose they looked for possible needle tracks, asked each respondent to demonstrate how he/she injected drugs, and to clarify specific 'slang' used by IDUs. All IDUs who passed this screening then provided informed consent. The study was approved by the regional medical-ethical committee (The Health Research Ethics Committee, Faculty of Medicine, Padjadjaran University/Dr. Hasan Sadikin General Hospital Bandung) and conducted within the context of program on prevention and treatment of HIV in the context of injecting drug use in Indonesia [13].

### Assessment

The interview was done at the community health center by trained interviewers who assured all participants that their anonymity would be strictly maintained. All participants who completed the interview session received a coupon for free HIV, HBV, HCV and syphilis testing at Hasan Sadikin hospital, Bandung. If found positive for HIV, participants were offered CD4-cell counts, chest X-ray and if needed, antiretroviral and/or syphilis treatment, all free of charge.

The interviewers used two validated questionnaires: the European Addiction Severity Index (EuropASI) and the Blood Borne Virus Transmission Questionnaires (BBV-TRAQ). The EuropASI is an adaptation of the Addiction Severity Index (fifth version). It is a semi-structured interview which takes about one hour, covering issues that may contribute to patients' substance-abuse problems, such as medical status, employment/support status, drug/alcohol use, legal status, family social relationship, and psychiatric problems [14]. Participants are asked if they ever used a number of listed drugs regularly (more than 3 times or 2 consecutive days a week). For regularly used drugs, further information is recorded including the first time the particular drug was used, the duration of use in a life time, the frequency of drug use in the previous 30 days, and drug route of administration [14]. ASI has shown excellent reliability and validity across a range of types of patients and treatment settings in many countries [15]. For the translation into Bahasa Indonesia, WHO translation procedures were used [16].

The BBV-TRAQ questionnaire assesses how often injecting drug users participate in specific injecting, sexual and other risk-practices that may expose them to blood-borne viruses. The instrument consists of 34 questions divided in three sub-scales which measure frequency of current risk behavior related to blood to blood transfer (20 questions); sexual practices (8 questions); and other skin penetration activities (6 questions) in the previous month. With respect to possible blood to blood transfer, information is collected about contact with contaminated needles and syringes, other drug injecting equipment sharing and involvement of other people in the drug preparation and injecting process. Questions related to sexual risk behavior address unprotected vaginal, anal, oral, and manual sex with other people, with or without lubricant, and during menstruation or not. Other questions address skin penetration risk behavior (tattooing and piercing), and shared use of toothbrush, razor, and personal hygiene equipment. The administration time for the instrument is short (around 15 minutes), and it has been shown good reliability and validity [17,18].

### Data analysis and statistics

A former IDU (fIDU) was defined as a person who reported to have injected an illicit drug at some point in his/her life, but not to have injected any drugs in the six months prior to the interview (17, 18). A current IDU (cIDU) was defined as a person who reported that he/she had injected any type of illicit drug in the six months prior to the interview [6,10]. Data were analyzed both descriptively and inferentially. Descriptive data are presented in terms of percentage, mean, and standard deviation. Subjects engaging in at least one risk taking behavior in a subscale of the BBV-TRAQ were regarded as taking risks in that domain. Data were analyzed inferentially for differences between fIDUs and cIDUs. Pearson Chi-Square was used for dichotomous data and the Mann-Whitney test for non-parametric continuous data. All tests were two-sided, with a P-value of 0.05 or less considered to indicate statistical significance. Analyses were performed with the use of SPSS, version 11.5.

## Results

### Characteristics of IDUs in Bandung

A total of 210 IDUs were recruited, of whom 194 were men (92%), 92 were fIDUs (44%), and 118 were cIDUs (56%). Thirty-three out of 92 fIDUs (35.9%) were invited by cIDUs, while 34 of 118 cIDUs (30.3%) were invited by fIDUs, showing extensive social linking between the two groups. Most of the demographic characteristics of fIDUs and cIDUs did not differ, except for the length of injecting drug and percentage of those who developed AIDS (table 1). The mean age was 28 (±4) years and most participants had graduated from senior high school and had been employed at some point in the last 3 years. They had started using drugs at a young age (14 (± 3) years). Injection of drugs had typically started 4 years after non-injecting drug administration, and the period of injecting drugs averaged 7 (± 4) years. Three quarters of the participants had been tested for HIV and among those tested 63% reported to be HIV-infected. There was no significant association between the cumulative years of drug injecting and HIV-status (P = 0.47). One third of those who were HIV-infected reported to have developed AIDS. Although cIDUs had injected for a longer period than fIDUs, HIV-infected individuals in the latter group more commonly reported having AIDS (44% vs 18%; P = 0,01).

**Table 1:** Sociodemographic characteristics of former and current injecting drug users

	Total group (N = 210)	fIDUs (N = 92)	cIDUs (N = 118)	P
Age, mean	27,8 (3,8)	28,1 (4,0)	27,5 (3,8)	0,64
Male gender	92%	89%	95%	0,12
Drug use				0,35
Age of first drug use, mean	14,0 (2,8)	14,2 (3,3)	13,8 (2,2)	0,31
Age of first drug injection, mean	18,0 (3,1)	18,4 (3,1)	17,8 (3,1)	0,17
Years of injecting in life time, mean	7,1 (3,8)	5,5 (3,6)	8,4 (3,4)	< 0,01
Marital Status				0,35
Married/remarried	30%	36%	25%	
Widowed	2%	3%	2%	
Separated/divorced	11%	6%	14%	
Never married	57%	55%	59%	
Employment in the past 3 years				0,13
Full time	41%	48%	36%	
Part-time	37%	36%	39%	
Student	5%	5%	4%	
Unemployed or housewife	17%	11%	21%	
Education				0,48
Junior high school or less	6%	4%	7%	
Senior high school	87%	87%	87%	
Undergraduate or higher	7%	7%	6%	
HIV-AIDS				
Ever HIV-tested	75%	71%	78%	0,23
HIV-infected^#^	63%	66%	60%	0,47
Have developed AIDS^##^	30%	44%	18%	0,01

All data are presented in percentage unless stated otherwise

^**# **^n = 145; no data available for 12 subjects; ^**## **^n = 83; no data for 8 HIV-positive subjects

The most used substance by all participants was heroin. Ninety four percent of the total participants had at some point used heroin regularly (at least three times a week or for two consecutive days in a week for more than 6 months). The other most used substances were cannabis, benzodiazepines, and alcohol. More than three quarters of participants had ever used or still used different drugs at the same time (poly drug use) and 70% of the total IDUs had ever used or still used amphetamine or methamphetamine regularly (median 2 years (range less than 1 year until 15 years).

In the last 30 days, cIDUs had typically used more drugs than fIDUs but neither cIDUs nor fIDUs reported total abstinence in the last 30 days. The most used substances by fIDUs in the last 30 days were alcohol, licit or illicit methadone/buprenorphine while cIDUs mostly used licit or illicit methadone/buprenorphine, heroin, benzodiazepines, and cannabis (table 2). Licit or illicit use of methadone or buprenorphin cannot be differentiated with the ASI.

**Table 2:** Drug use among former and current injecting drug users

**Kind of drug **^**#**^	Life time drug use			Drug use in last 30 days		
	
	fIDUs (n = 92)	cIDUs (n = 118)	P	fIDUs (n = 92)	cIDUs (n = 118)	P
Any use of alcohol	91%	97%	0,14	42%	59%	0,03
Alcohol, over threshold ^##^	66%	70%	0,66	26%	42%	0,02
Heroin	99%	100%	0,44	0%	79%	< 0,01
Methadone or buprenorphine	33%	65%	< 0,01	12%	53%	< 0,01
Other opiates	22%	23%	0,87	1%	5%	0,14
Benzodiazepines	66%	79%	0,06	12%	52%	< 0,01
Amphetamine	52%	53%	0,89	1%	9%	0,03
Cannabis	84%	87%	0,55	13%	47%	< 0,01
Ecstasy (MDMA)	44%	37%	0,40	9%	14%	0,28
More than one substance	78%	83%	0,38	12%	55%	< 0,01

^# ^regular use (more than 3 times or 2 consecutive days a week)^. ## ^≥ 3 drinks in 1-2 hours, ≥ 3 times or 2 consecutive days a week.

Note: Less than three participants used inhalant, hallucinogens or cocaine in the last 30 days.

### Risk behavior related to transmission of blood-borne viruses

Blood to blood transfer risk behavior was reported by 76% of cIDUs and 13% of fIDUs (X^2 ^= 82,73; p < 0,01) (table 3). Risk behavior of ninety cIDUs (76%) was related to sharing of contaminated drug injecting equipment. Current IDUs often reported behavior associated with a very high risk of transmission of blood-borne pathogens, including injecting with another person's used needle or syringe (reported by 15% of cIDUs), re-use of a needle or syringe taken out of a shared disposal/sharps container without using bleaching (9%), and sharp injuries from another person's used needle/syringe (15%). cIDUs also reported behavior associated with a somewhat lower risk of transmission. For example, they had exposure to contaminated drug injecting equipment included shared use of a tourniquet (43%); injecting a drug prepared with water previously used by another person (41%); handling another person's used needle or syringe when wounded at his or her hand (34%); wiping his/her own injection site with an object that had been used by another person (27%); touching his/her own injection site soon after 'assisting' another person with their injection (26%); and injecting a drug that was prepared immediately after 'assisting' another person with their injection but without washing hands between activities (26%). While fIDUs did not inject anymore, 13% still had some risk of blood borne pathogens transmission, especially through accidental needle stick injuries and sucking or licking and other handling of another person's used needle/syringe. Interestingly, benzodiazepine use was more common among IDUs engaging in risky injecting behavior, 56% vs. 39% among cIDUs (X^2 ^= 2,26; P = 0,19) and fIDUs (33% vs 9%; X^2 ^= 5,99; P = 0,03). Injecting risk behavior was not associated with use of alcohol, cannabis or methadone/buprenorphin (data not shown).

**Table 3:** Risk behavior among former (fIDUs) and current injecting drug users (cIDUs) in the last 30 days

Risk Behavior	fIDUs (N = 92)	cIDUs (N = 118)	**X**^**2**^	P
Blood-blood transfer, n (%)	13%	76%	82,73	<0,01
Suck or lick a filter which had been used by another person	4%	14%	5,81	0,02
Inject a drug prepared with water which had been used by another person	0%	41%	45,30	<0,01
Been injected by another person who had already injected or assisted in someone else's injection	0%	20%	21,13	<0,01
Receive an accidental needle-stick/prick from another person's used needle/syringe	3%	15%	7,56	<0,01
Re-use a needle/syringe taken out of a shared disposal/sharps container	0%	9%	8,26	<0,01
Sexual risk behavior, n (%)	42%	56%	3,57	0,06
Engage in unprotected vaginal sex with another person	35%	47%	2,98	0,08
Engage in unprotected vaginal sex with another person during menstruation	11%	20%	2,90	0,09
Engage in unprotected anal sex with another person	7%	8%	0,10	0,76
Tattoo or piercing, n (%)	52%	53%	0,02	0,90
Tattooed by someone who was not a professional tattooist	4%	15%	6,56	<0,01
Been pierced by someone who was not a professional piercer	10%	14%	0,96	0,327
Use another person's toothbrush	3%	14%	7,45	<0,01

Sexual risk behavior of some form was reported by 56% of cIDUs and 42% of fIDUs (X^2 ^= 3,57; P = 0,06). The most common sexual risk behavior for cIDUs and fIDUs was unprotected vaginal sex (47% respectively 35%, NS); reported unprotected anal sex was much lower (8% respectively 7%, NS). No statistical significant differences were found between former and current IDUs regarding oral and manual sex (data not shown). Excessive alcohol use was more common in IDUs engaging in risky sexual behavior, both among cIDUs (51% vs 33%; X^2 ^= 3,86; P = 0,06) and fIDUs (46% vs 11%; X^2 ^= 14,14; P < 0.01). Other risk behavior such as tattooing and piercing, which confer a much lower risk of HIV transmission compared to needle-sharing, was reported by half of all respondents (52% of fIDUs and 53% of cIDUs).

## Discussion

This cross-sectional study from Indonesia shows a high prevalence of HIV-infection among relatively young and well-educated former and current IDUs. Current IDUs obviously had much higher risks of viral transmission related to injecting drugs, but also former IDUs had risks related to blood to blood transmission. Importantly, both groups engaged in substantial risks related to sexual transmission of HIV. Almost half of respondents in our study were former IDUs, showing that this group may contribute significantly to HIV transmission.

Injecting drug use is the main factor driving the HIV-epidemic in Indonesia. In a large cohort of HIV patients in our setting, two thirds had a history of IDU [19]. In line with this finding, the prevalence of HIV-infection among fIDUs and cIDUs in this study was high (66% respectively 60%), similar or slightly higher compared with previous local and national data [4]. The prevalence of AIDS was higher among fIDUs; concern about their general health or the development of AIDS may cause them to move from injecting drugs [6].

Injecting with needles or syringe from other people (15%) or from shared disposal/sharps containers without bleaching (9%) was reported by a substantial proportion of IDUs, although lower compared to previous research in Indonesia (24% - 80%) [5,20]. The high rate of needle stick injuries is no surprise given the fact 40% of IDUs fail to discard used needles safely [21]. Risk related to injecting drugs was especially high in cIDUs, but fIDUs are experience certain things (e.g. needle sticks or sharing of injecting equipment) which may have a low risk of HIV-transmission, but which may pose a significiant risk of transmitting HCV. This is important, as the prevalence of HCV is very high among IDUs in this setting; among 633 HIV patients with a history of IDU 87.7% were HCV-infected [19].

Both among current and former IDUs, sexual risk behavior may contribute significantly to HIV transmission. Sexual risk behavior was equally high in both groups, which is in contrast with previous studies reporting associations between injecting drug use and unsafe sex [22,23]. Condom use among IDUs has found to be inconsistent and especially low with sex workers and other risk groups for HIV transmission [20]. As IDUs often have multiple sex partners, including sex workers, HIV transmission may easily spread to people outside the IDU community.

Heroin was the most frequently used drug among IDUs in this sample but many reported use of cannabis, benzodiazepines and alcohol as well, in line with reports from China, Thailand, Ukraine, Lithuania, and Poland [24]. The use of methadone or buprenorphine among cIDUs was higher compared to fIDUs. The EuropASI questionnaire does not allow us to verify whether this was as part of substitution treatment or not. Both drugs are officially registered in Indonesia for substitution treatment but illegal use is quite common. Buprenorphine injection has been reported in many countries including Indonesia, and can be regarded as a response to inadequate care, rather than simply as misuse [25].

Alcohol abuse was common, and was associated with risky sexual behavior, as has been reported previously [26]. The same has been reported for methamphetamine and amphetamine use [27], but this seems still relatively rare in this setting. Our study also showed a high prevalence of tattooing and piercing in former and current IDUs, both of which have may lead to transmission of HIV and viral hepatitis [8,9,28].

This study suffers form the limitations of a cross-sectional study in a population which is difficult to reach, and the question is therefore how representative the samples are. By using RDS, we tried to minimize this risk [12,29]. Numerical simulations have shown that the possible bias, even if the seeds are not drawn randomly, is extremely small (0.3%) for all sample sizes greater than 200 [12]. Still, some IDUs who are not in the social networks with these participants can not be recruited through respondent driven sampling [30].

## Conclusions

In conclusion, this study from Indonesia shows that former IDUs, when compared to current IDUs, may have similar high HIV prevalence rates and high sexual risk behavior. Specific programs focusing on the reduction of sexual risk behavior are needed among former and current IDUs, in order to prevent further transmission to the general community. Drug-use treatment and interventions that examine the relationship between drug use and sexuality should be conducted. In addition, earlier HIV treatment may lower transmission among IDUs by reducing the 'community viral load'[31]. We have recently shown that patients with a history of injecting drug use in our setting have a similar clinical and virological response to anti-retroviral treatment compared to non-IDUs [19]. Finally, evidence-based prevention should also be targeted at schoolchildren and young adolescents as injecting drug use starts at an early age in this setting.

## Competing interests

The authors declare that they have no competing interests.

## Authors' contributions

SI made and carried out the research protocol, performed the statistical analysis, and drafted the manuscript. DB and LP participated in making the research protocol. TH and IS participated in the design of the study. RvC, AvV, CdJ conceived of the study, and participated in its design and coordination and helped to draft the manuscript. All authors read and approved the final manuscript.

## Pre-publication history

The pre-publication history for this paper can be accessed here:

http://www.biomedcentral.com/1471-2458/10/472/prepub
